# The Impact of Flash Glucose Monitoring on Markers of Glycaemic Control and Patient Satisfaction in Type 2 Diabetes

**DOI:** 10.7759/cureus.16007

**Published:** 2021-06-28

**Authors:** Ayman Al Hayek, Mohamed Al Dawish, Manal El Jammal

**Affiliations:** 1 Department of Endocrinology and Diabetes, Prince Sultan Military Medical City, Riyadh, SAU; 2 Scientific Affairs, Abbott Diabetes Care, Dubai, ARE

**Keywords:** diabetes mellitus, flash glucose monitoring, freestyle libre®, glycaemic control, intermittent scanning continuous glucose monitoring, treatment satisfaction, type 2 diabetes

## Abstract

Introduction: The effect of flash glucose monitoring on glycaemic control and patient satisfaction in insulin-treated type 2 diabetes (T2D) from Saudi Arabia is uncertain. The aim of this prospective observational study was to evaluate the change in HbA1c (Hemoglobin A1c) and satisfaction with treatment following the initiation of flash glucose monitoring.

Methods: This single-arm, single-centre prospective observational study included flash glucose monitoring-naive adult patients with T2D managed with multiple daily injections of insulin therapy (MDI) and HbA1c ≥7%. HbA1c was measured, and the Diabetes Treatment Satisfaction Questionnaire (DTSQ, Arabic version) and Glucose Monitoring Satisfaction Survey (GMSS) were completed at baseline and 12 weeks.

Results: For participants (n=54) from one diabetes centre, HbA1c significantly improved by 0.44% from 8.22%±0.69 (mean±SD) at baseline to 7.78%±0.71 at 12 weeks, p<0.001. Confirmed hypoglycaemic episodes reduced from 4.43±1.51 episodes/month to 1.24±1.15 (-3.19, p<0.001). Glucose monitoring frequency improved, indicated by the number of scans per day, with a mean increase of 5.13 (p <0.001) tests/day. GMSS scores improved across all four categories, as did overall treatment satisfaction (p<0.001 for all categories). Patients perceived clear improvements across all questions relating to satisfaction and frequency of hypo- or hyperglycaemic episodes.

Conclusion: Following initiation of flash glucose monitoring in patients with T2D and MDI insulin therapy, HbA1c improved with reduced hypoglycaemic events and increased patient-reported satisfaction. This study contributes valuable data on the use of flash glucose monitoring in this population, and a larger multicentre study is warranted to inform future health policy for T2D in Saudi Arabia.

## Introduction

Type 2 diabetes mellitus (T2D) is a growing public health problem globally, posing a significant challenge to healthcare systems. The onus of management rests largely upon the individual patient, and this can present a significant burden. Self-management encompasses broad factors such as diabetes medication adherence, diet, physical activity, and blood glucose self-monitoring in achieving optimal glycaemic control. Physical, social, and mental distress are well characterised phenomena in patients with diabetes, and these can impact the ability to self-manage. A number of tools are available to make various aspects of diabetes management easier for patients and hence to improve health and psychosocial outcomes. One such tool is continuous glucose monitoring (CGM), which has grown in popularity in recent years, overcoming some of the inconveniences of traditional finger-prick methods. The FreeStyle Libre® flash glucose monitoring system (Abbott Laboratories, Chicago, IL, USA) measures interstitial glucose levels via a small sensor applied to the back of the upper arm and has been described in detail elsewhere [1]. On scanning the sensor, discrete real-time glucose readings are provided. A number of studies on the use of flash glucose monitoring in T2D have emerged globally, supporting the notion that the system contributes to improvements in self-management in terms of both glycaemic control and quality of life [2-8]. However, the use of flash glucose monitoring remains poorly characterised in patients with T2D in the Arab region and in Saudi Arabia in particular. Saudi Arabia was placed seventh in global rankings of T2D prevalence and second within the Middle East in a 2005 report by the World Health Organization [9]. A more recent report from the International Diabetes Federation places the Middle East and North Africa region first in regional rankings of world-age standardised diabetes prevalence [10]. This continued upward trend in the pervasiveness of T2D is accompanied by evidence of poor glycaemic control in this patient group [11, 12], which has sparked calls for more comprehensive nationwide research to enable tailoring of self-management programs to meet the needs of the T2D population in Saudi Arabia [13, 14]. In order to begin the process of addressing this data paucity, the primary aim of this prospective observational study was to introduce flash glucose monitoring to patients with T2D managed with multiple daily injections of insulin therapy (MDI), currently using self-monitoring of blood glucose (SMBG), and evaluate the impact on HbA1c, treatment satisfaction, and glucose monitoring satisfaction.

## Materials and methods

Study design

This prospective, observational, single-arm study was conducted over 12-weeks in a single diabetes centre in Riyadh, Saudi Arabia. Consecutive patients were invited to participate if they were; aged 20-75 years, had a diagnosis of T2D, an HbA1c ≥7% (or fasting  blood  glucose ≥110 mg/dL, <250 mg/dL), prescribed an MDI insulin regimen for at least one year, used conventional SMBG to test their glucose levels at least two times per day, had no prior experience of flash glucose monitoring or any other CGM system, and attended regular follow-up appointments at our centre. Individuals were not included if they had; a history of pancreatitis, severe infections, severe mental illnesses, or malignant disease; history of serious vascular diseases (such as stroke or myocardial infarction) within six months prior to the initiation of the study, pregnancy, or planned future pregnancy or were deemed as unfit to participate by the primary physician.

Patients attended the diabetes treatment centre for their baseline and follow-up visits as per the study protocol. Additional appointments were arranged as per the centre protocol for follow-up with the treating physician and educator.

At the baseline visit, the FreeStyle Libre sensor was attached to the back of the patient’s upper arm by a trained diabetes educator. Patients were educated and trained on the proper application of the sensor and followed instructions as per the label. Patients were advised that, as far as possible, current glucose levels should be checked by scanning the sensor at least every eight hours. In addition, participants were advised to confirm sensor glucose levels with an SMBG test during unsteady glucose states, during impending or suspected hypoglycaemia, and if their sensor readings did not match their symptoms. Demonstrations were given on how sensor glucose levels could be confirmed with a capillary measurement using the blood glucose meter in-built in the reader of the flash glucose monitoring system. No additional counselling or education was provided. However, all participants were given the contact details of the diabetes educator, who could be contacted at any time during the study. At the end of the study, sensor data were downloaded to a computer to produce the reports, including the ambulatory glucose profile, in order to identify the number of scans performed during the study period.

At the baseline visit, demographic characteristics were recorded (age, sex, height and weight, and duration of diabetes) at baseline and the 12-week visits clinical characteristics (frequency of SMBG or flash glucose monitoring scans per day, number of SMBG confirmed hypoglycaemic episodes per month, the total daily dose of insulin and HbA1c) were recorded. HbA1c was measured using the COBAS INTEGRA 400 plus/800 analyzers at the central laboratory of the Prince Sultan Military Medical City (PSMMC). Baseline clinical data for frequency of SMBG for the preceding four weeks and frequency of hypoglycaemic events were collected using the FreeStyle Optium Neo® blood glucose meter (Abbott Laboratories, Chicago, IL, USA) and FreeStyle Auto-Assist Neo® software.

Participants completed the Arabic versions of the Diabetes Treatment Satisfaction Questionnaire - Status Version (DTSQs) at baseline and at 12 weeks the Diabetes Treatment Satisfaction Questionnaire - Change Version (DTSQc, [15-17]). Participants also completed the Glucose Monitoring Satisfaction Survey (GMSS) version T2D at the baseline and study end visits (12 weeks). This questionnaire is focused particularly on satisfaction with the mode of glucose monitoring. Responses give rise to four summary scores: emotional burden, behavioural burden, openness, and worthwhileness, where higher scores indicated greater perceived levels of these parameters [18].

Ethics

Participants gave written informed consent to participate in the study. The study protocol was approved by the Research Ethics Committee of the PSMMC in accordance with the Helsinki Declaration of 1964, revised in 2013 (ethical approval no. 1394-HP-01-R079). All participants could withdraw at any point without reason or prior notice.

Statistical analysis

Summarized parameters were expressed as mean ± standard deviation (SD) and range. The statistical significance of changes between patients’ paired baseline and 12-week data was determined by way of the two-tailed Student’s t-test or the Wilcoxon-Mann-Whitney U test, depending on outcomes of normality testing of timepoint differences (𝛼 = 0.05), and were expressed as mean ± standard deviation and 95% confidence interval (CI). The associations between clinical parameters such as HbA1c, frequency of daily monitoring, and rate of hypoglycaemic episodes were assessed by visual inspection of scatter plots and calculation of Pearson’s product-moment correlation coefficient. The association between changes in clinical parameters and treatment satisfaction was calculated in a similar fashion. Additional exploratory analysis was conducted, particularly on the association of age and scan frequency. All statistical analysis was conducted in R version 4.0.1.

Outcomes

The primary outcomes were changes in HbA1c levels, patient-reported diabetes treatment satisfaction, and patient-reported glucose monitoring satisfaction from baseline to 12 weeks. Secondary outcomes included the change in the number of SMBG confirmed hypoglycaemic episodes per month, change in body mass index (BMI), change in the weight-to-height ratio (WtHr), and change in frequency of glucose monitoring per day.

## Results

All subjects (n=54) were recruited from the Diabetes Treatment Center, Prince Sultan Military Medical City, Riyadh, Saudi Arabia, between October 2019 and May 2020. Participant demographics and baseline characteristics are shown in Table 1. 

**Table 1:** Demographics and baseline characteristics of the study population (n=54).

Characteristic		n (%)
Gender	Female	26 (48%)
	Male	28 (52%)
Age in years	Overall mean 41.6 (range, 29–55)	
	25–34	8 (15%)
	35–44	30 (55%)
	45–55	16 (30%)
Body mass index (BMI)kg/m^2^	Overall mean 31.5	
	BMI <25	2 (4%)
	25≤ BMI <30	14 (26%)
	BMI ≥30	38 (70%)
Waist-to-height ratio (WtHR)†	WtHR <0.5	1 (2%)
	0.5≤ WtHR <0.6	22 (41%)
	WtHR ≥0.6	31 (57%)
Duration of diabetes in years	Overall mean 3.4 (range 1-6 years)	
	<4 years	29 (54%)
	≥4 years	25 (46%)
Baseline HbA1c	8.22% (mean)	
Total daily dose of insulin	1.45 ± 0.35 (units/kg/day)	
Frequency of glucose monitoring per day	Overall mean 2.48	
	2	31 (57%)
	3	20 (37%)
	4	3 (6%)
Frequency of hypoglycaemic episodes per month	Overall mean 4.43 ± 1.51	
	2–4	28 (52%)
	5–7	25 (46%)
	>7	1 (2%)

A statistically significant improvement was observed in HbA1c at 12 weeks, which fell by 0.44% (p< 0.001) from 8.22% ± 0.69 (mean ± SD) from 7.78 ± 0.71. There was a greater absolute drop in HbA1c in participants with a higher HbA1c at baseline. Moreover, those with higher baseline BMI (>30) experienced a greater absolute drop in HbA1c as well as a greater drop relative to baseline HbA1c measures (BMI >30, absolute ΔHbA1c = -0.47%, relative ΔHbA1c = -5.54%; BMI <30, absolute ΔHbA1c = -0.39%, relative ΔHbA1c = -4.45).

At baseline the number of confirmed hypoglycaemic episodes experienced varied from two to eight per month) which fell by a mean of 3.19 episodes per month (p<0.001, Table 2). Total daily dose on insulin fell by 0.43 units/kg/day (p<0.001, Table 2).

Participants performed significantly more sensor scans per day, compared to SMBG frequency at baseline, with a mean increase of 5.13 monitoring episodes per day (p <0.001, Table 2)

**Table 2:** Outcome of paired, two-tailed statistical testing of baseline against 12-week clinical parameters, where (*) indicates P-values exceeding the prespecified 𝛼 of 0.05. (SD: standard deviation.)

Measure	Baseline mean ± SD (range)	12-week mean ± SD (range)	Mean difference	P-value	95% confidence interval
HbA1c% (range)	8.22 ± 0.69 (7.2–11.1)	7.78 ± 0.71 (6.2–9.9)	-0.44	<0.001*	0.40, 0.60
Confirmed hypoglycaemic episodes per month (range)	4.43 ± 1.51 (2–8)	1.24 ± 1.15 (0–4)	-3.19	<0.001*	2.64, 3.73
Total daily dose of insulin Units/kg/day (range)	1.45 ± 0.35 (0.9–2.1)	1.02 ± 0.26 (0.6–1.7)	-0.43	<0.001*	0.35, 0.55
Frequency of glucose monitoring per day (range)	2.48 ± 0.60 (2–4)	7.61 ± 1.73 (4–12)	5.13	<0.001*	4.50, 5.50
Body mass index (kg/m^2^) (range)	31.5 ± 3.04 (24.4–37.9)	30.7 ± 2.93 (24.1–37.5)	-0.80	<0.001*	0.61, 0.99
Waist-to-height ratio (WtHr) (range)	0.61 ± 0.06 (0.49–0.73)	0.60 ± 0.05 (0.49–0.73)	-0.01	<0.001*	0.01, 0.02

**Figure 1:**
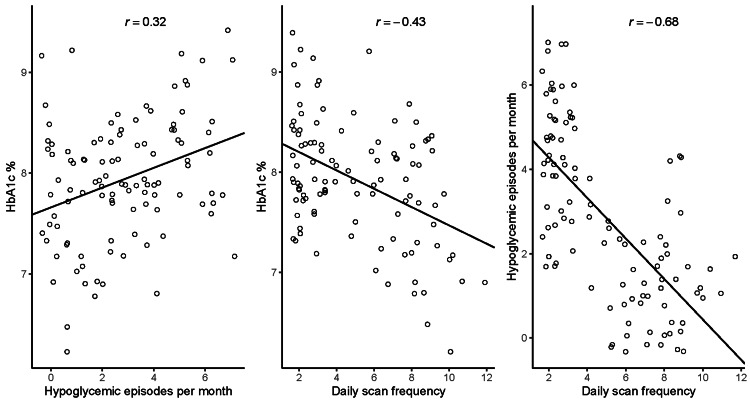
Linear relationships between glycated haemoglobin (HbA1c) level, frequency of hypoglycaemic episodes per month and daily scan frequency, over the course of the study. Lines of best fit were calculated by least squares method. Pearson’s product-moment correlation coefficients are indicated by r.

In correlation analysis, linear relationships were found between HbA1c level and daily scan frequency (r = -0.43, p <0.001), between the frequency of hypoglycaemic episodes per month and daily scan frequency (r = -0.68, p <0.001), and between HbA1c level and the frequency of hypoglycaemic episodes per month (r = 0.32, p <0.001, Figure 1).

Exploratory analysis revealed that mean scan frequency did not significantly differ when we split the cohort into three age bands (25-34, 35-44, and >45 years), either at baseline or at 12 weeks.

The majority of participants experienced weight loss. The overall change in mean BMI was modest (p<0.001, Table 2).

Patient-reported satisfaction questionnaires

**Figure 2:**
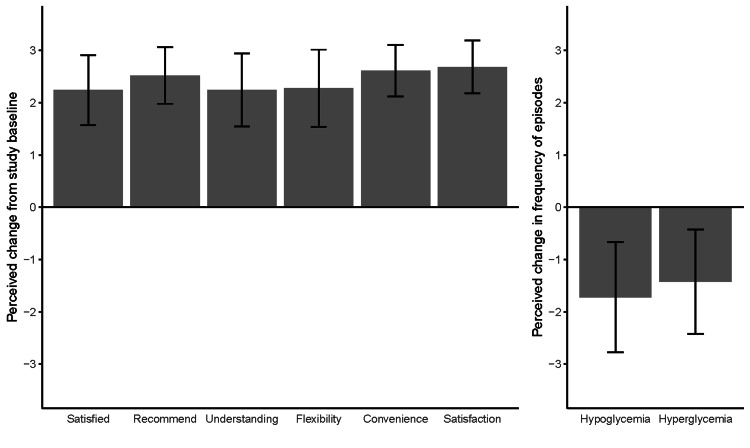
Results of the diabetes treatment satisfaction questionnaire – change version (DTSQc), expressed as mean change from study baseline ± standard deviation (error bars).

Outcomes of DTSQs completed by all subjects at baseline, expressed as mean ± standard deviation, are summarised as follows. The combined total treatment satisfaction score was 14.1 ± 2.56 (range, 9-20; a range of possible scores, 0-36). The combined score of perceived episodes of hypo- or hyperglycaemia was 7.33 ± 1.52 (range, 4-10; a range of possible scores, 0-12). Outcomes of DTSQc questionnaires are summarised in Figure 2, with clear perceived improvements across all questions relating to satisfaction relative to baseline. Outcomes of DTSQc also clearly evidence a reduction in the perceived rates of hypo- or hyperglycaemic episodes.

**Figure 3:**
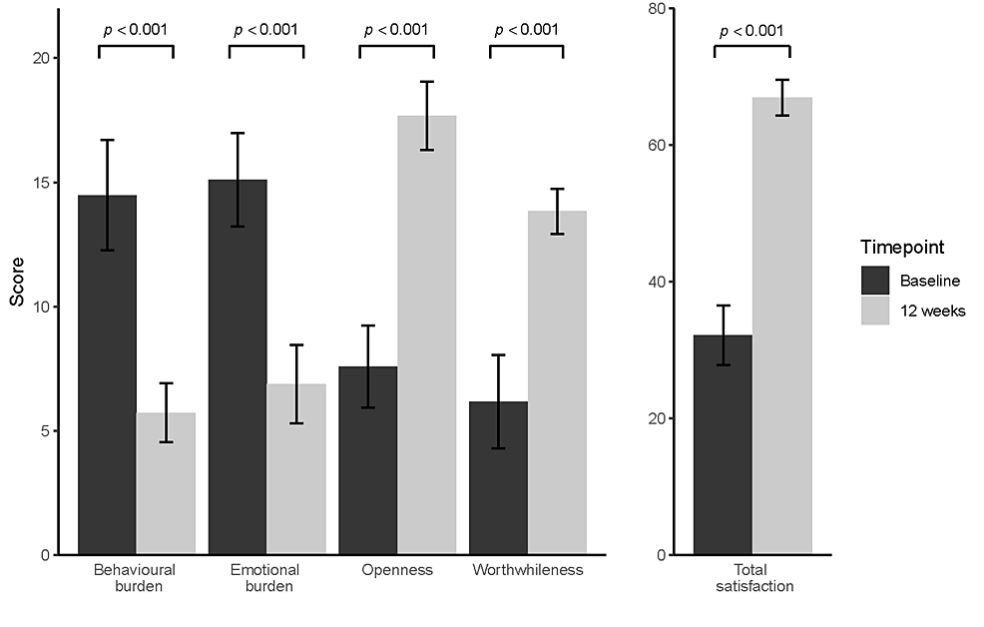
Outcomes of glucose monitoring satisfaction survey (GMSS) at baseline and 12 weeks, expressed as mean aggregated score ± standard deviation (error bars).

Outcomes of the GMSS are given in Figure 3. Two-fold, statistically significant improvements were experienced from baseline to 12 weeks across all four categories of behavioural burden (14.5 ± 2.23 vs. 5.72 ± 1.19, p <0.001, CI: 8.00, 9.50), emotional burden (15.1 ± 1.88 vs. 6.87 ± 1.58, p <0.001, CI: 7.50, 9.00), openness (7.59 ± 1.65 vs. 17.7 ± 1.37, p <0.001, CI: 9.50, 11.0) and worthwhileness (6.17 ± 1.87 vs. 13.8 ± 0.91, p <0.001, CI: 7.50, 8.00). Similarly, the total treatment satisfaction score doubled over the course of the study (32.2 ± 4.39 vs. 66.9 ± 2.63, p <0.001, CI: 33.5, 36.0). A negative linear correlation was identified between HbA1c levels and GMSS total treatment satisfaction (r = -0.32, p<0.001), and similar correlation trends were found between HbA1c levels and the four summary categories of the GMSS.

## Discussion

In this study, we introduced flash glucose monitoring to a cohort of patients with T2D treated with MDI therapy who had previously only used a traditional finger-prick method of blood glucose monitoring. We have shown that the introduction of flash glucose monitoring was associated with improvements in markers of glycaemic control and self-reported patient satisfaction, as well as decreases in perceived and actual episodes of hypoglycaemia, the total daily dose of insulin, BMI, and WtHr, despite a relatively short study duration. As far as we are aware, this represents first-time data on the use of flash glucose monitoring in the Saudi Arabian T2D population. Similar studies conducted in Saudi Arabia to date are limited to type 1 diabetes [19, 20]. While these support our present findings on the positive effects of switching to flash glucose monitoring, T2D is of course a distinct entity with unique aetiologies and thus demands its own body of evidence that is apposite to Saudi Arabia. Our findings are supported by a number of observational studies, registries, and a limited number of RCTs (Randomized controlled trial) conducted elsewhere [2-8]. The REPLACE trial found significant HbA1c reductions in a pre-specified subgroup of patients under 65 years of age over a six-month period, and the switch to flash glucose monitoring resulted in a significant reduction in time spent in hypoglycaemia and significantly fewer hypoglycaemic episodes per day [3]. More recently, a reduction in HbA1c was demonstrated in a similar population of T2D managed with MDI therapy in a longer RCT from Israel and in three European chart review studies [3, 4]. In a recent meta-analysis of the safety and efficacy of flash glucose monitoring by Castellana et al (2020), which included RCTs as well as prospective and retrospective cohort studies of both type 1 diabetes and T2D, the technology was deemed an effective strategy in diabetes management [21]. Furthermore, in a real-world European analysis of over 60 million glucose tests, Dunn et al (2018) identified reductions in estimated A1c with the use of flash glucose monitoring [5]. While it has been reported that for each percentage increase in mean initial HbA1c, (for example above 7%), the expected mean change in final HbA1c falls by 0.31% [22], the observed reduction in HbA1c should be interpreted cautiously as a similar reduction was reported for the control arm of an RCT in the same population [3]. Similarly, the identified trends between BMI and HbA1c change are hypothesis-generating and remain to be explored further. The need to understand which clinical factors represent significant drivers of HbA1c reduction in the broader Saudi population is ever-present and could be explored in a larger randomised controlled trial. The reason for the observed improvement in glycaemic control in the present study is unclear. The mean total daily dose of insulin decreased from baseline to study end and most subjects lost a modest amount of weight, with differences in the paired analysis found to be statistically significantly different from baseline to 12 weeks. Speculatively, these changes may be a consequence of clinical review and participants’ lifestyle modifications, either independently or in combination. In particular, weight loss is potentially a positive benefit from decreased insulin doses. However, during the study, these activities were supported by more comprehensive flash glucose data which suggests that this was a factor and this rationale is supported by recent studies [2-4]. Furthermore, hypoglycaemic episodes decreased from baseline to study end which also makes the use of comprehensive glucose data to support insulin dose requirements and titration at specific times of day more likely.

These observations of improved HbA1c with a reduction in hypoglycaemic episodes are notable in a population with T2D managed with MDI therapy. Furthermore, the modest change in BMI is clinically important and highly pertinent. The prevalence of obesity in the Saudi population is a major concern as it has continued to increase and it is estimated that up to 7 out of every 10 people could be classified as obese [23, 24]. In the current study, the vast majority of participants were overweight or obese, with a WtHr in the suggestive of risk or high risk (>0.6) categories (Table 1, [25]). Recently reported data suggests there is a strong association between WHtR and cardiac risk in individuals from Saudi Arabia. As it is established that T2D already carries an increased risk for cardiovascular morbidity and mortality [26], the potential of weight loss in a population with T2D deserves further investigation in a future study [27].

In the present study cohort, reductions in HbA1c were experienced fairly systematically by all subjects. Those with higher baseline HbA1c experienced greater reductions in HbA1c, both absolutely and as a proportion of baseline measures, which has been reported by others [3, 4]. HbA1c reduction was associated with a reduction in episodes of hypoglycaemia, as well as an increase in the frequency of glucose monitoring with the number of scans carried out by patients. This observation supports real-world data in European populations [5]. Daily glucose monitoring at baseline was low compared to a European cohort [2] and similar to the previously reported frequency of glucose monitoring for Saudi Arabia [28]. Monitoring frequency increased with the use of flash glucose monitoring in this study population with MDI therapy which has been observed previously in Europe [2] and in type 1 diabetes in Saudi Arabia [19, 20]. Also encouraging is the finding that flash glucose monitoring scan frequency in the current study did not vary by age group, which supports the notion that this technology device is easy to use.

From our present study, which included measurements of patient-reported satisfaction, we surmise that convenient access to their interstitial glucose measures might be empowering for patients. The observed scanning rate which was three times that of SMBG frequency at baseline would seem to support this rationale. A recent meta-analysis by Cowart et al (2020) of RCTs studying the impact of flash glucose monitoring in both type 1 diabetes and T2D identified greater patient satisfaction and lower diabetes distress with flash glucose monitoring as compared with usual care [29]. Our study identified correlations between measures of patient satisfaction and clinical measures, which serve as cross-validity as well as underscoring the central importance of patients’ wellbeing with regard to adherence to treatment regimens. This is particularly pertinent in diabetes, where issues of societal stigma are still prevalent and pervasive, accentuated by the fact that the burden of management rests upon the patients themselves.

This study was subject to a number of limitations that should be acknowledged. The cohort of participants was small and the study was conducted within a single diabetes centre. As such these results may not reflect possible outcomes at different centres or regions of Saudi Arabia. However, the baseline HbA1c and mean age of participants is similar to that of the large Middle Eastern cohort in the Global HAT study [30] which may support the generalisability of our findings. This was a relatively short study and it is not known if the reductions in HbA1c, hypoglycaemic episodes, and weight loss would be maintained over a longer period. The present study provides novel evidence from standard clinical care settings in Saudi Arabia, however, the observational methodology limited recording of certain clinical parameters, such as more detailed glucose metrics and percentage time in ranges, which would have added further interest. In future studies, it would be useful to know how frequently clinical reviews took place, what medication adjustments were made, and what glycaemic changes occurred with an analysis of sensor data. It would also be valuable to understand how patients’ perceptions of quality of life, as well as clinical changes, are sustained or indeed exceeded over a greater period of follow-up. Yaron et al (2019) provide evidence that additional counselling throughout the study period may improve the modification of lifestyle risk factors [3].

This study experience could be valuable to contribute to the design of a clinical trial. A future longer-term study could include RCT methodology and characterisation of all glycaemic metrics and lifestyle behaviours. Such a study would provide valuable insights into individualising management strategies to meet the unique needs of the T2D MDI population in Saudi Arabia.

## Conclusions

This single centre, 12-week study provides valuable, novel data on the use of flash glucose monitoring in patients with T2D and MDI therapy in Saudi Arabia. The findings were an improved HbA1c and reduction in hypoglycaemic episodes with increased satisfaction with treatment after switching to flash glucose monitoring. The inclusion of this glucose monitoring technology to support modern management of T2D may have the potential to ease some of the social and economic burden of diabetes management in Saudi Arabia. The finding of significant improvements in glycaemic control and patient satisfaction after switching to flash glucose monitoring would benefit from validation in a larger multi-centre study in order to inform future health policy for the growing population of patients with T2D.
